# Gender disparities in comprehensive knowledge of HIV among adolescents and young adults in Ethiopia: A decomposition analysis

**DOI:** 10.1038/s41598-026-43778-0

**Published:** 2026-03-09

**Authors:** Mekdes Tamiru Yizengaw, Samrawit Birhanu Alemu, Aynalem Belay, Melaku Birhanu Alemu

**Affiliations:** 1 St. Lideta Health Science College, Addis Ababa, Ethiopia; 2 Department of Public Health, Debre Markos University, Debre Markos, Ethiopia; 3 Department of Midwifery, Wolkite University, Wolkite, Ethiopia; 4 Department of Health Systems and Policy, Institute of Public Health, University of Gondar, Gondar, Ethiopia; 5 Curtin School of Population Health, Curtin University, Perth, WA Australia

**Keywords:** Patient education, Public health

## Abstract

Female adolescents and young adults (AYAs) aged 15–24 bear up to a threefold higher burden of HIV and face nearly twice the susceptibility to infection compared with males of the same age group. A comprehensive knowledge of HIV is critical for effective prevention of the virus. However, substantial gender disparities persist in HIV knowledge. This study assessed male and female differences in comprehensive HIV knowledge among AYAs in Ethiopia and identify key drivers of these disparities. This study used a cross-sectional design with secondary data analysis of the 2016 Ethiopian Demographic and Health Survey (EDHS). Male and female AYAs aged 15 to 24 were included in the study. Comprehensive HIV knowledge was the outcome variable. Explanatory variables included age, educational attainment, marital status, occupation, household wealth, media exposure, and region of residence. Sample weighting was applied. Descriptive statistics and multivariate decomposition analysis were conducted to assess gender disparities. The decomposition model separated differences into explained (endowments) and unexplained (coefficient effects) components. A total of 10,597 AYAs (6,143 females and 4,455 males) were included in the analysis. Only 30.5% (95% CI: 29.6, 31.4) of AYAs demonstrated comprehensive HIV knowledge, with markedly higher levels among male AYAs (39.1%) compared with female AYAs (24.33%). Urban residents had higher HIV knowledge than their rural counterparts, males (52.3%) and females (58.3%). Education and occupation were significant contributors to the knowledge difference between males and females, with individuals who had higher education or were employed showing better HIV knowledge. Internet use and media exposure also played a crucial role, with those exposed demonstrating significantly higher HIV knowledge. Gender disparities in HIV knowledge were primarily driven by education, occupation, and region. There are significant gender disparities in HIV knowledge among AYAs in Ethiopia, with males generally exhibiting higher levels of knowledge than females. The findings highlight the need for targeted HIV education programs that address these gender gaps, particularly for young women, who are more vulnerable to HIV infection. Socioeconomic factors, including education, residence, and media exposure, play a crucial role in shaping HIV knowledge. Efforts to enhance HIV knowledge through gender-sensitive interventions are crucial for improving prevention efforts and ultimately reducing HIV incidence in Ethiopia and similar contexts.

## Introduction

Globally, around 40.8 million people are living with HIV/AIDS, with 1.3 million new infections and 630,000 fatalities reported in 2024^1^. The epidemic has had its greatest impact in SSA, which emerged as the central area of concern for HIV transmission during that period^2^. Women and girls across all age groups continue to bear a disproportionate share of the HIV burden in the region, accounting for approximately 63% of all new HIV infections. An estimated 4,000 adolescent girls and young women aged 15–24 years acquired HIV each week globally, nearly 3,300 of whom were in sub-Saharan Africa^1^.

In 2023, an estimated 1.9 million female AYAs 15–24 years were living with HIV, compared with 1.2 million adolescent boys and young men of the same age group, highlighting a substantial gender disparity in HIV burden among young people^3^. Approximately 85% of HIV cases are reported in SSA, where the HIV epidemic has a disproportionately high impact. This is largely attributed to factors such as restricted access to healthcare, inadequate education, and limited availability of prevention services^4^.

Female AYAs aged 15–24 have up to three times the likelihood of living with HIV and twice the susceptibility to the infection compared to males in the same age group^5,6^. This could be as a result of biological factors, such as increased vulnerability to bodily fluids during sexual activity, as well as gender disparities, social discrimination, limited autonomy in sexual and reproductive decisions, intimate partner violence, inadequate access to reproductive healthcare, and restricted educational and economic opportunities^6,7^.

The high rate of new infections can be partially linked to insufficient comprehensive knowledge regarding HIV/AIDS and the available prevention strategies^8^. Insufficient HIV knowledge among AYAs, along with socio-cultural influences, may play a role in fostering stigma toward individuals who are infected or affected by HIV^9^. Males exhibit a higher level of HIV knowledge than females. This disparity suggests a need for targeted educational interventions to enhance HIV awareness among young women^10^. AYAs have access to a variety of information sources to improve their understanding of HIV, including family, peers, educators, and online platforms. Having sufficient knowledge about HIV is essential for safeguarding AYAs, as research indicates they are one of the most at-risk groups^11^. Socioeconomic characteristics such as age, marital status, education level, occupation, wealth index, and media exposure were found to be significant determinants of comprehensive knowledge of HIV^12,13^.

Comprehensive knowledge of HIV has been examined in several studies, with prior work documenting its overall prevalence and associated sociodemographic determinants^14,15^. Existing evidence has also highlighted wealth-related inequalities in comprehensive HIV knowledge^16^. However, despite well-documented gender disparities in HIV vulnerability among AYAs, there is a clear lack of evidence quantifying gender inequalities in comprehensive HIV knowledge among AYAs and, critically, identifying the factors contributing to these disparities.

We hypothesise that there is a statistically significant gender disparity in comprehensive knowledge of HIV among adolescents and young adults (15–24 years) in Ethiopia, with males having higher levels of comprehensive HIV knowledge than females. In addition, the observed gender disparity in comprehensive HIV knowledge is partially explained by differences in sociodemographic characteristics (endowments), such as education, household wealth, media exposure, and place of residence, and partially attributable to differential effects of these characteristics between males and females (coefficient effects).

## Methods

### Study design

This study is a secondary analysis of cross-sectional data from the 2016 Ethiopian Demographic and Health Survey (EDHS). The EDHS is a nationally representative survey conducted between January and June 2016, designed to provide estimates of key demographic and health indicators at national and subnational levels. For the present analysis, we restricted the analytic population to AYAs aged 15–24 years, consistent with the study objective of examining gender disparities in comprehensive HIV knowledge within this age group. The analysis reflects HIV knowledge among Ethiopian AYAs at the time of the 2016 EDHS^17^.

### Data source

The EDHS 2016 data, which were intended to provide estimates of indicators for the nation, for urban and rural populations separately, for the nine regions, and for the two-city administrative divisions, were used in this study’s secondary data analysis. Public access to the EDHS dataset can be found at https://dhsprogram.com/data/ available-datasets. cfm.

### Sampling procedure

A sampling frame from the 2015 Population and Housing Census (PHC) in Ethiopia is used by the Central Statistical Agency (CSA) to provide EDHS 2016. A comprehensive list of all census enumeration areas (EAs) is included in the census frame. There are 84,915 EAs in all. An EA typically includes 181 homes, which are initially divided into 202 urban and 443 rural households based on the size of the EA. Then, from the newly generated household listing, 28 households per cluster were selected using a systematic random sampling process^18^. A total of 16,583 women were eligible for individual interviews, and 15,683 completed the process. The study utilised individual records from the EDHS dataset, which included 15,683 women of reproductive age. Additionally, data from male participants were used, with 14,795 eligible men identified in the sampled households, of whom 12,688 were successfully interviewed^18^.

### Study population and analytic sample

This secondary analysis utilised the individual recode (IR) for female data and the male recode (MR) datasets from the EDHS. The initial sample comprised all interviewed women aged 15–49 years (IR file) and men aged 15–59 years (MR file). For the purposes of this study, the analytic population was restricted to adolescents and young adults aged 15–24 years.

**Fig. 1:**
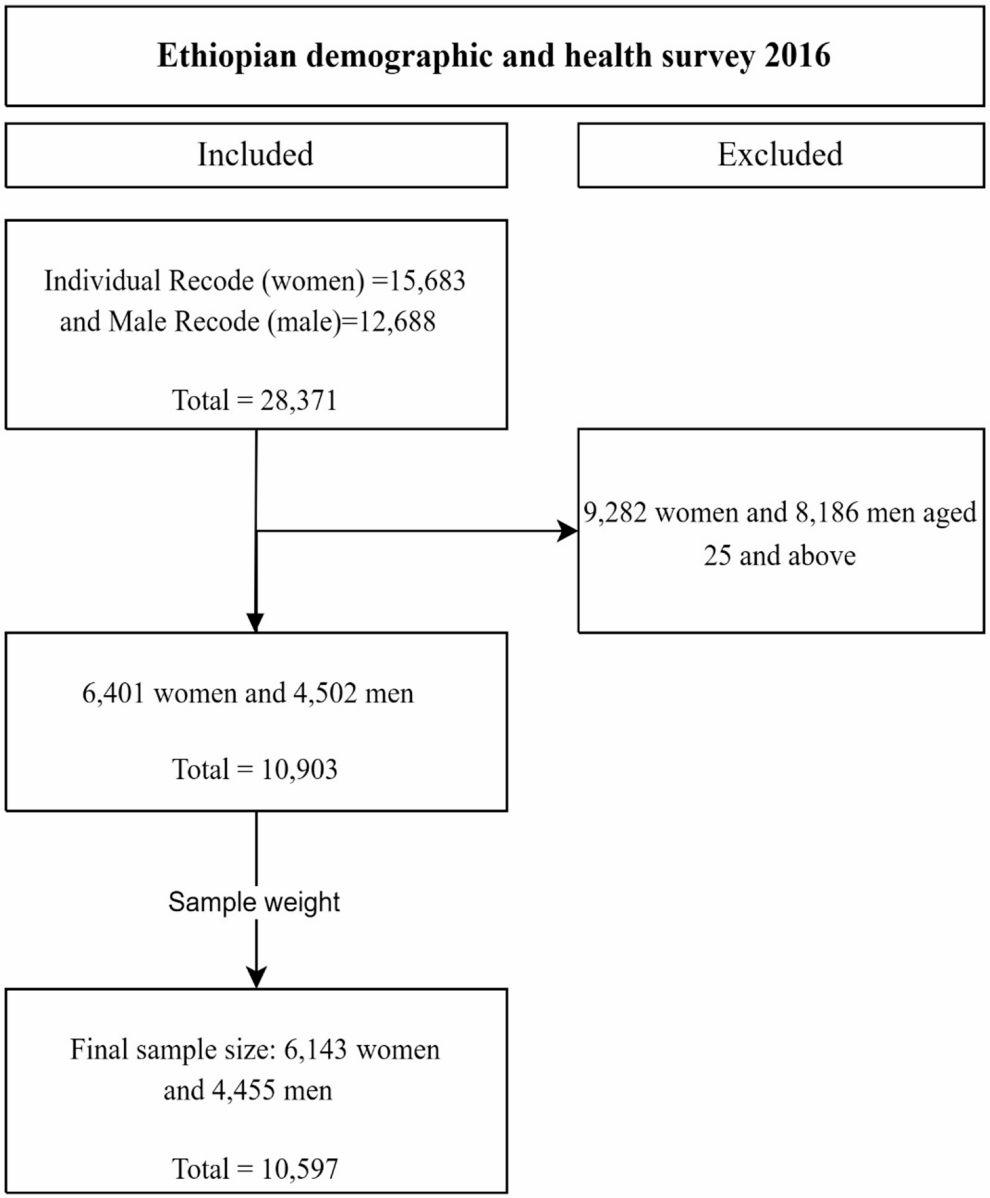
Flow diagram of sample selection for adolescents and young adults (15–24 years) from the 2016 Ethiopian Demographic and Health Survey.

Participants were included if they (1) were aged 15–24 years at the time of the survey, (2) had complete information on the comprehensive HIV knowledge variables, and (3) had valid responses for all covariates included in the decomposition analysis. Respondents aged 24 years and above, as well as those with missing or incomplete data on the outcome variable, were excluded. The unweighted sample comprised 6,401 females and 4,502 males, for a total of 10,903 adolescents and young adults. After applying sampling weights, the weighted sample comprised 6,143 females and 4,455 males, yielding a final sample size of 10,597 (Fig. 1).

## Measurements of variables

### Dependent variable

Comprehensive HIV knowledge was assessed using five standard DHS items that evaluated knowledge on the transmission and prevention of HIV. Respondents were classified as having comprehensive HIV knowledge if they correctly identified that a healthy-looking person could have HIV, rejected the misconceptions that HIV can be transmitted through mosquito bites or by sharing food, and correctly recognised that consistent condom use and having one faithful, uninfected partner reduce the risk of HIV transmission. Each item was recoded as binary, and respondents who answered all five items correctly were coded as having comprehensive HIV knowledge; all others were coded as lacking comprehensive HIV knowledge^19^.

### Independent variables

In this study, all independent variables were treated as covariates of comprehensive HIV knowledge and were selected based on existing literature and data availability in the EDHS. Given the descriptive and decomposition-based nature of the analysis, variables were not classified as confounders within a causal inference framework. Instead, they were included to quantify their contribution to observed gender differences in HIV knowledge through differences in characteristics (endowments) and effects (coefficients). Sex served as the grouping variable in the decomposition analysis, and no additional effect modification was formally tested.

Age was categorised as 15-19 as “Adolescents^15–19^”, and 20 to 24 as " Young adults^20–22^. Marital status was recoded into two categories: married and unmarried. The region was categorised as “central” (Tigray, Amhara, Oromia, SNNP), “peripheral” (Afar, Gambela, Benishangul, Somalia), and “metropolises” (Addis Ababa, Harari and Dire Dawa). Occupation was recoded as “yes” (working AYAs) and “No " (non-working). Media exposure was categorised as “yes” if respondents had access to at least one media source (newspaper, radio, or television) at least once per week, and “no” otherwise^20^.

### Data management and analysis

Prior to analysis, the IR and MR datasets were separately cleaned and restricted to respondents aged 15–24 years, then analysed by sex. Sampling weights provided by the EDHS were applied to account for the complex survey design, including stratification and clustering, and to restore national representativeness of estimates for the adolescent and young adult population.

Descriptive statistics were used to summarise the socioeconomic and demographic characteristics of the study participants. Frequencies and percentages were presented using tables and figures to illustrate the distribution of these characteristics and the prevalence of comprehensive HIV knowledge across key subgroups.

Multivariate decomposition analysis is used to assess the extent to which the gender gap in average predicted outcomes is explained by factors included in multivariate models. Specifically, the difference in comprehensive HIV knowledge between males and females can be broken down into two main components: differences in the distribution of characteristics (endowments) and differences in the influence of those characteristics (coefficients).

Statistical significance was assessed at the 5% level, and results were reported at the 95% confidence interval. All analyses were conducted using Stata version 17.

### Missing data handling

Comprehensive HIV knowledge was constructed from five standard DHS items. Following the standard DHS approach, “don’t know” and missing responses were coded as lack of knowledge for each item. Covariates were harmonised across gender using standard DHS definitions. Analyses were conducted using complete-case observations, and the final sample comprises all eligible respondents with the required non-missing information for outcome construction and model estimation.

## Results

### Characteristics of the participants

A total of 10,597 AYAs aged 15–24 years were included in this study. Approximately 56% of the participants were adolescents aged 15–19. Of the respondents, 59% were female. Most of the AYAs (57%) attended primary school, while approximately 17% had not received any formal education. A majority of the AYAs (78%) resided in rural areas. The majority of the AYAs (44%) identified as Orthodox Christians, followed by Muslims (Table 1).

**Table 1:** Socioeconomic and demographic characteristics of respondents in 2016 (*n* = 10,597).

Variables		Male *n* = 4,455	Female *n* = 6,143	Total *n* = 10,597
Age	Adolescent	2,572(57.7)	3,381(55.0)	5,953(56.2)
	Young adult	1,883(42.3)	2,762(45.0)	4,645(43.83)
Marital status	Unmarried	3,955(88.8)	3845 (62.6)	7,800(73.6)
	Married	500(11.2)	2,298(37.4)	2,798(26.4)
Residence	Rural	3,588(80.5)	4,675(76.1)	8,263(78.0)
	Urban	867(19.5)	1,467(23.9)	2,335(22.0)
Religion	Orthodox	2,007(45.5)	2,640(43.0)	4,647(43.9)
	Muslim	1,339(30.1)	1,883(30.7)	3,222(30.4)
	Protestant	1,013(22.7)	1,487(24.2)	2,500(23.6)
	Others	96(2.2)	133(2.2)	229(2.2)
Region	Central	4,013(90.1)	5,360(87.3)	9,373(88.5)
	Peripheral	210(4.7)	327(5.3)	537(5.1)
	Metropolises	232(5.2)	456(7.4)	688(6.5)
Education	No education	543(12.2)	1,230(20.0)	1,773(16.7)
	Primary education	2,744(61.6)	3,333(54.3)	6,076(57.3)
	Secondary education	910(20.4)	1,184(19.3)	2,095(19.8)
	Higher	258(5.8)	396(6.4)	654(6.2)
Wealth	Poorest	735(16.5)	1,015(16.5)	1,750(16.5)
	Poorer	810(18.2)	1,153(18.8)	1,963(18.5)
	Middle	855(19.2)	1,275(20.8)	2,130(20.1)
	Richer	957(21.5)	1,294(21.1)	2,251(21.5)
	Richest	1,099(24.7)	1,405(22.9)	2,504(23.6)
Occupation	Yes	3,644(81.8)	2,691(43.8)	6,336(59.8)
	No	811(18.2)	3,451(56.2)	4,262(40.2)
Use- internet	No	3,725(83.6)	5,679(92.5)	9,404(88.7)
	Yes	730(16.4)	464(7.6)	1,194(11.3)
Age at First sex	No	3,340(75.0)	3,292(53.6)	6,632(62.6)
	Yes	1,115(25.0)	2,851(46.4)	3,966(37.4)
Media- exposure	Yes	2,938(66.0)	3,104(50.5)	6,042(57.0)
	No	1,517(34.1)	3,039(49.5)	4,556(43.0)

### Comprehensive knowledge of HIV

**Fig. 2:**
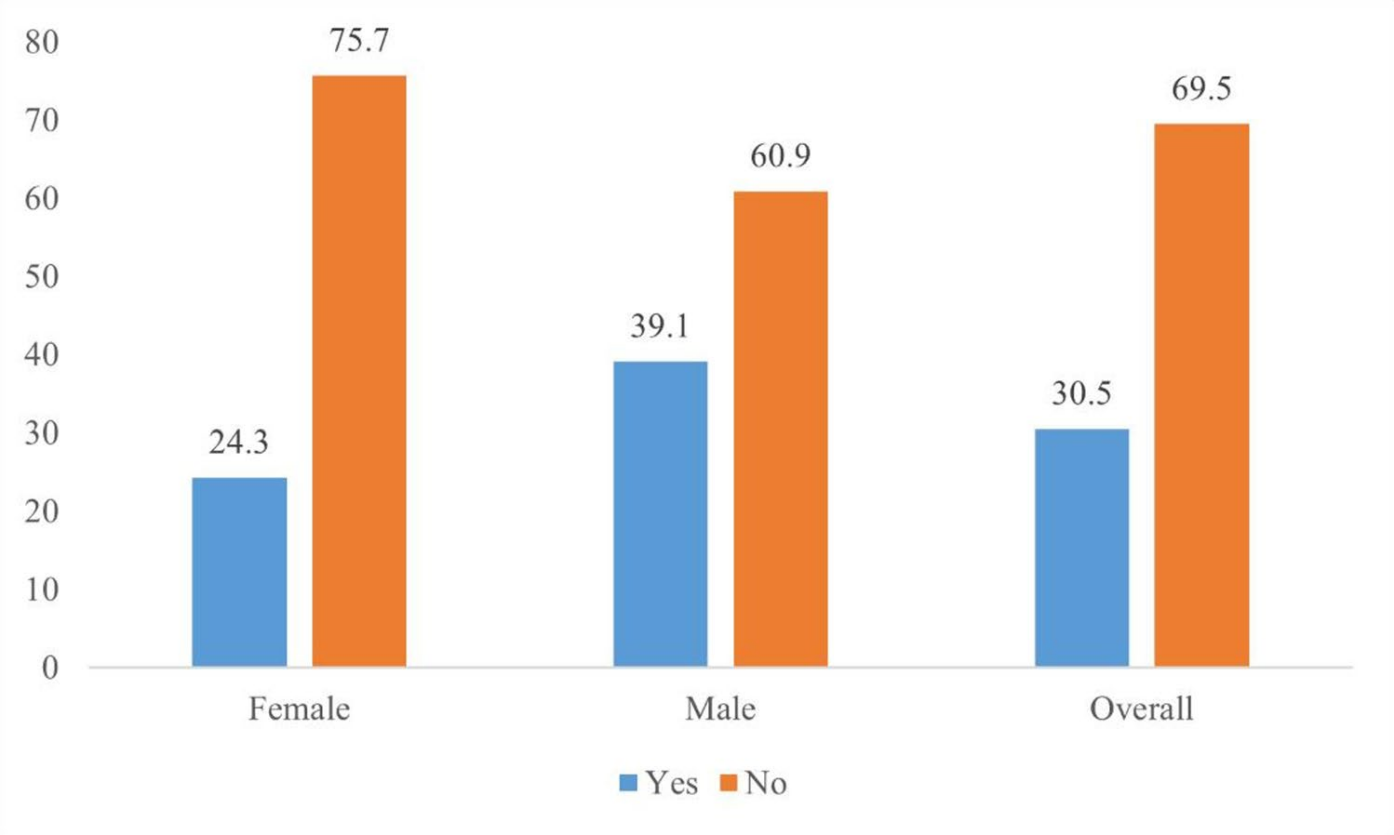
Comprehensive knowledge of HIV among adolescents and young adults by gender.

Comprehensive knowledge of HIV among adolescents and young adults (AYAs) in Ethiopia was 30.5% (95% CI: 29.6, 31.4). However, substantial gender disparities were observed: only 24.3% (95% CI: 23.2, 25.4) of female AYAs demonstrated comprehensive knowledge, compared with 39.1% (95% CI: 37.6, 40.5) among male AYAs. Female AYAs had significantly lower comprehensive HIV knowledge than their male counterparts (*p* < 0.05) (Fig. 2).

When stratified by age, 31.2% of young adults and 29.9% of adolescents demonstrated comprehensive HIV knowledge. Notable gender disparities persisted across all age groups. Among adolescents, 37.6% of males had comprehensive knowledge compared with 24.0% of females. A similar pattern was observed among young adults, where 41.1% of males demonstrated comprehensive knowledge, compared with 25.0% of females. Similarly, unmarried males had slightly higher levels of comprehensive HIV knowledge (42.3%) than married males (39.3%). Among females, the gap was more pronounced: 32.0% of unmarried females had comprehensive knowledge compared with only 20.7% of married females. Overall, differences across all sociodemographic characteristics were statistically significant (Table 2).

**Table 2:** Comprehensive knowledge of HIV among adolescents and young adults by gender, EDHS 2016.

Variable	Category	Men (*n* = 4,455)		Women (*n* = 6,143)		Total (*n* = 10,598)		Chi 2 test
		No	Yes	No	Yes	No	Yes	
Age group	Adolescent	1,605(62.40)	967(37.60)	2,568 (75.96)	813 (24.04)	4,173 (70.10)	1,780 (29.90)	0.010
	Young adult	1,110 (58.95)	773 (41.05)	2,083 (75.41)	679 (24.59)	3,193 (68.74)	1,452 (31.26)	
Marital status	Married	313.4 (62.65)	187 (37.35)	1,867 (81.24)	431 (18.76)	2,180 (77.92)	618 (22.08)	0.000
	Unmarried	2,402 (60.73)	1,553 (39.27)	2,784 (72.40)	1,061 (27.60)	5,186 (66.48)	2,614 (33.52)	
Religion	Orthodox	1,103 (54.95)	904 (45.05)	1,843 (69.82)	797 (30.18)	2,946 (63.40)	1,701 (36.60)	0.000
	Muslim	884 (66.02)	455 (33.98)	1,554 (82.53)	329 (17.47)	2,438 (75.67)	784 (24.33)	
	Protestant	666 (65.78)	347 (34.22)	1,132 (76.11)	355 (23.89)	1,798 (71.93)	702 (28.07)	
	Other	62 (64.37)	34 (35.63)	122 (91.47)	11 (8.53)	184 (80.09)	46 (19.91)	
Residence	Urban	454 (52.32)	414 (47.68)	856 (58.32)	612 (41.68)	1,310 (56.09)	1,025 (43.91)	0.000
	Rural	2,261 (63.03)	1,326 (36.97)	3,795 (81.17)	881 (18.83)	6,056 (73.29)	2,207 (26.71)	
Region	Central	2,432 (60.59)	1,582 (39.41)	4,070 (75.93)	1,290 (24.07)	6,502 (69.36)	2,872 (30.64)	0.000
	Peripheral	173 (82.22)	37 (17.78)	292 (89.49)	34 (10.51)	465 (86.65)	72 (13.35)	
	Metropolises	111 (47.76)	121 (52.24)	289 (63.27)	168 (36.73)	399 (58.04)	289 (41.96)	
Education	No education	395 (72.83)	147 (27.17)	1,128 (91.64)	103 (8.36)	1,523 (85.88)	250 (14.12)	0.000
	Primary	1,721 (62.73)	1,023 (37.27)	2,620 (78.62)	713 (21.38)	4,341 (71.44)	1,735 (28.56)	
	Secondary	491 (53.89)	420 (46.11)	710 (59.93)	475 (40.07)	1,200 (57.31)	894 (42.69)	
	Higher	108 (41.85)	150 (58.15)	193 (48.89)	202 (51.11)	302 (46.11)	352 (53.89)	
Wealth index	Poorest	499 (67.98)	235 (32.02)	850 (83.77)	165 (16.23)	1,350 (77.14)	400 (22.86)	0.000
	Poorer	503 (62.11)	307 (37.89)	928 (80.41)	226 (19.59)	1,431 (72.86)	533 (27.14)	
	Middle	494 (57.73)	361 (42.27)	918 (72.02)	357 (27.98)	1,412 (66.28)	718 (33.72)	
	Richer	606 (63.39)	350 (36.61)	952 (73.53)	343 (26.47)	1,558 (69.22)	693 (30.78)	
	Richest	613 (55.75)	486 (44.25)	1,003 (71.39)	402 (28.61)	1,616 (64.52)	888 (35.48)	
Occupation	No	556 (68.54)	255 (31.46)	2,656 (76.96)	795 (23.04)	3,212 (75.36)	1,050 (24.64)	0.000
	Yes	2,159 (59.25)	1,485 (40.75)	1,994 (74.10)	697 (25.90)	4,154 (65.56)	2,182 (34.44)	
Internet use	No	2,348 (63.03)	1,377 (36.97)	4,417 (77.77)	1,263 (22.23)	6,764 (71.93)	2,640 (28.07)	0.000
	Yes	367 (50.29)	363 (49.71)	234 (50.47)	230 (49.53)	601 (50.36)	593 (49.64)	
Media exposure	No	1,075 (70.89)	442 (29.11)	2,582 (84.97)	457 (15.03)	3,658 (80.28)	899 (19.72)	0.000
	Yes	1,640 (55.81)	1,298 (44.19)	2,068 (66.64)	1,035 (33.36)	3,708 (61.37)	2,334 (38.63)	

^1^Catholic, traditional, and others.

### **Decomposition of gender disparities in comprehensive knowledge of HIV**

This study attributed the gender disparities in comprehensive knowledge of HIV to 43% compositional factors (endowments) and 57% coefficient effects (Table 3).

**Table 3:** Overall decomposition of gender disparities in comprehensive knowledge of HIV.

Endowment	Coefficient	β (Std. Err)	Per cent	*P* value
	0.06	0.00928	42.99	0.000
Coefficient	0.08	0.01235	57.01	0.000

A significant variation in participants’ composition was observed by region, religion, education, occupation, internet usage, residence, and media exposure (Table 4).

For the endowments (E) effect, occupation showed the largest contribution, accounting for 27.47% of the gender disparity in comprehensive knowledge of HIV. Media exposure and primary education also contributed substantially, at 8.75% and 4.10%, respectively. Secondary education contributed 1.07%, while being Protestant contributed 0.84%, and being Muslim added 0.22%. Peripheral region residence contributed 0.73%. In contrast, higher education had a negative contribution (−0.91%).

For the coefficients (C) effect, rural residence had the most substantial positive contribution, explaining 36.12% of the disparity, followed by occupation at 28.24%. Living in metropolitan regions also showed a positive contribution of 3.64%. However, education played a strong negative role, with primary and secondary education contributing − 24.69% and − 14.02%, respectively. Being Protestant contributed − 6.70%.

**Table 4:** Contributing factors for gender disparities in comprehensive knowledge of HIV, EDHS 2016.

Variable	Category	Endowments (E)		Coefficients (C)		
		β (Std. Err)	Percent (%)	β(Std. Err)		Percent (%)
Age in years	15–19	Ref				
	20–24	0.00000 (0.00039)	0.00	−0.01065(0.00778)		−7.21
Current marital status	Unmarried	Ref				
	Married	0.00032 (0.00580)	0.22	−0.01695(0.01429)		11.48
Religion	Orthodox	Ref				
	Muslim	0.00033 (0.00009) ***	0.22	−0.00273(0.00578)		−1.85
	Protestant	0.00124 (0.00024) ***	0.84	−0.00989 (0.00468) **		−6.70
	Other ^1^	0.00000 (0.00000)	0.00	0.00331 (0.00140)		2.24
Region	Central	Ref				
	Peripheral	0.00107 (0.00024) ***	0.73	−0.00118(0.00246)		−0.80
	Metropolises	−0.00004 (0.00071)	−0.03	0.00538(0.00253) **		3.64
Education	No education	Ref				
	Primary education	0.00605(0.00156) ***	4.10	−0.03645(0.01391) ***		−24.69
	Secondary education	0.00158(0.00029) ***	1.07	−0.02071(0.00569) ***		−14.02
	Higher	−0.00135(0.00023) ***	−0.91	−0.00422(0.00266)		−2.86
Residence	Urban	Ref				
	Rural	−0.00124(0.00096)	−0.84	0.05333(0.01753) **		36.12
Wealth	Poorest	Ref				
	Poorer	−0.00005(0.00013)	−0.04	0.00468(0.00513)		3.17
	Middle	−0.00055(0.00035)	−0.37	−0.00277(0.00551)		−1.87
	Richer	−0.00008(0.00009)	−0.05	−0.00555(0.00565)		−3.76
	Richest	0.00057(0.00040)	0.39	0.00234(0.00617)		1.59
Occupation	No	Ref				
	Yes	0.04055(0.00652) ***	27.47	0.04170(0.00824) ***		28.24
Use-internet	No	Ref				
	Yes	0.00215(0.00195)	1.46	−0.00206(0.00204)		−1.40
Media_ exposure	No	Ref				
	Yes	0.01292(0.00223) ***	8.75		0.00108(0.00885)	0.73

^1^Catholic, traditional, and others.

## Discussion

The findings of this study provide critical insights into the gender disparities in comprehensive HIV knowledge among AYAs in Ethiopia and how different socio-demographic and structural factors play a role in these differences. Comprehensive HIV knowledge among AYAs in Ethiopia was 30.5% (95% CI: 29.6, 31.4). Females had significantly lower knowledge than males (24.3% compared with 39.1%).

These disparities were also evident across age groups. Among males, 38% of adolescents (15–19 years) and 41% of young adults (20–24 years) demonstrated comprehensive HIV knowledge, whereas the corresponding proportions among females were substantially lower at 24% and 25%, respectively. These disparities are consistent with previous studies from SSA, and a study conducted in Uganda^21,10^. This may be due to males often having greater access to education, media, and public health messages. In addition to this, females may face difficulties in discussing sex and sexual information with their peers and family members because discussions on such issues are socially unacceptable^22^.

The analysis reveals that 43% of the differences are due to compositional differences, whereas 57% are due to differences in effects. The composition differences in sociodemographic characteristics such as education, occupation and residence significantly explained the gender disparities in comprehensive knowledge of HIV. This finding was in line with a study assessing wealth-related inequalities in comprehensive knowledge of HIV in Ethiopia^16^. In addition, more than half of the gender disparity in comprehensive HIV knowledge was attributable to differences in effects, indicating that similar socioeconomic characteristics do not translate into equivalent gains in knowledge for males and females. This pattern is consistent with the heterogeneity observed in adolescents’ and young adults’ preferences for sexual and reproductive health service attributes, where the same structural conditions may be valued and experienced differently across males and females^23^.

Educational attainment significantly contributes to HIV knowledge disparities. Similar findings were reported in Nigeria^24^. This may be because females may have less access to continued education, limiting their exposure to health education. In addition to this, females, often facing educational barriers, may be less likely to receive comprehensive information about HIV prevention and treatment, leading to lower knowledge levels compared to their male counterparts^25^.

Occupation (28%) is a substantial contributor, highlighting a significant role in shaping access to and understanding of HIV-related information, as individuals in different jobs or employment statuses likely have varying levels of exposure to health education. Employment may expose individuals, particularly males, to health education sessions, especially in the formal sector. This may be unemployed or informally employed women who are frequently excluded from institutional HIV education programs, which can have serious consequences for their health and well-being^26^.

Media access (8.75%) has been found to contribute to the gender disparity in comprehensive knowledge of HIV, indicating that unequal exposure to information channels plays a substantive role in shaping knowledge gaps between male and female adolescents and young adults. This contribution suggests that males are more likely than females to have regular access to mass media. Males often have more access to mobile phones and the internet, increasing their exposure to sexual and reproductive health information. This aligns with findings from Kenya^27^ and Ethiopia^28^, which showed that internet and media access are strongly correlated with HIV knowledge, especially among males.

### Limitations of the study

This study has some limitations that must be acknowledged. First, the data were cross-sectional, limiting the ability to establish causal relationships between variables. Additionally, the reliance on self-reported data may have introduced reporting biases, particularly regarding sensitive topics such as sexual activity and HIV knowledge. Given that the analysis is based on the 2016 EDHS, the most recent survey capturing comprehensive HIV knowledge, the findings may not fully reflect recent trends. Furthermore, the study does not explore the deeper underlying socio-cultural factors that contribute to the observed gender disparities in HIV knowledge, which may warrant further qualitative research. Potential sources of bias include nonresponse, misclassification of self-reported HIV knowledge, and gender-related differences in reporting, which could not be fully addressed within the scope of this secondary analysis.

## Conclusion

This study highlights significant gender differences in comprehensive HIV knowledge among AYAs in Ethiopia, with males generally demonstrating a higher level of awareness than females. The findings suggest that gender disparities in HIV knowledge are influenced by various socio-economic and demographic factors, including education, residence, and media exposure. Given the higher vulnerability of young women to HIV, targeted educational interventions focused on increasing HIV awareness among this group are essential. These programs should aim to address barriers such as limited access to education and healthcare, and the cultural factors that contribute to unequal HIV knowledge.

Targeted and gender-responsive HIV education strategies are urgently needed in Ethiopia. Interventions should prioritize female AYAs by expanding access to formal education and inclusive media and digital platforms, particularly for those who are unemployed, informally employed, or out of school. Community- and school-based programs should be strengthened to address sociocultural norms that limit open discussion of sexual health for females, with active engagement of families, religious leaders, and community stakeholders to ensure culturally sensitive delivery. Addressing both structural inequalities and differential returns to education and employment is essential to narrowing gender disparities in comprehensive HIV knowledge and improving HIV prevention outcomes among Ethiopian AYAs.

## Data Availability

The dataset is available on the Measure DHS website: https://dhsprogram.com/.
